# Infertility policy analysis: a comparative study of selected lower middle- middle- and high-income countries

**DOI:** 10.1186/s12992-020-00617-9

**Published:** 2020-10-23

**Authors:** Bahar Morshed-Behbahani, Minoor Lamyian, Hassan Joulaei, Batool Hossein Rashidi, Ali Montazeri

**Affiliations:** 1 Department of Reproductive Health and Midwifery, Faculty of Medical Sciences, Tarbiat Modares University, Tehran, Iran; 2 Department of midwifery, School of Nursing & Midwifery, Shiraz University of Medical Sciences, Shiraz, Iran; 3 Health policy Research Center, Institute of Health, Shiraz University of Medical Sciences, Shiraz, Iran; 4 Vali-e-Asr Reproductive Health Research Center, Faculty of Medicine, Tehran University of Medical Sciences, Tehran, Iran; 5 Population Health Research Group, Health Metrics Research Center, Institute for Health Sciences Research, ACECR, Tehran, Iran; 6 Faculty of Humanity Sciences, University of Sciences & Culture, ACECR, Tehran, Iran

**Keywords:** Policy analysis, Infertility care, Universal health coverage

## Abstract

**Background:**

Infertility has recently become a salient but neglected global issue. Policies to address the sexual and reproductive health and rights (SRHR) are vital, especially in lower middle and middle-income countries (LMICs). Hence, the aim of this study was to compare the national infertility policies in the selected countries (LMICs comparing with high-income) to determine gaps or to confirm desirable policies in the given health systems.

**Methods:**

This study has executed a comparative policy analysis of infertility services using the universal health coverage framework (financial protection, population coverage, and service features) in three scopes (prevention, treatment, and supportive care). Seven countries that had infertility programs in their health sectors were selected.

**Results:**

The results showed that financial protection was good in high and middle-income countries, but in a lower middle income, and in one high-income country was poor. The findings also showed that health systems in the same countries had no infertility services for men. Preventive and supportive care services were neglected in LMICs by governments.

**Conclusion:**

The findings indicate that income is not the only factor that fulfills universal health coverage for infertility care services. Perhaps to achieve equity in infertility care services, it should be seen as a universal human right to accomplish the right to have a child and to have a life with physical and mental health for all men and women.

## Introduction

Infertility is a condition described by the failure to establish a clinical pregnancy after 12 months of orderly and unprotected sexual relations, which is an important public health issue [1]. Globally, more than 186 million people suffer from infertility [2]. It can deeply influence people’s lives, causing medical, social, economic, and psychological harm [3, 4]. The World Health Organization (WHO), has announced that ‘Infertility generates disability (impairment of function), and thus access to healthcare falls under the Convention on the Rights of Persons with Disability’ [5]. Currently WHO has employed a greater urgency on ‘prevention’ as a key policy to address the global burden of infertility and adopted a tertiary care level approach for infertility [6]. The recently published” National Public Health Action Plan for the Detection, Prevention and Management of Infertility” by the Centers for Disease Control and Prevention (CDC) in the United State of America has called for better access to excellent infertility services and enhanced safety of fertility treatments [7].

Despite these efforts, considerable gaps and opportunities exist in surveillance, research, communication, program, and policy development on infertility care in health systems. In recent years, the prevalence of infertility has increased due to postponement of childbearing (in developed countries), sexually transmitted infections (STI), harmful deliveries and unsafe abortion (in developing countries) [2, 8, 9]. In many countries due to the high cost of infertility treatment, many infertile couples go untreated or undertreated. Many of them have to pay out of pocket for their medical treatment because either they do not have health insurance or their insurance policies do not include fertility care [3]. There are few countries that have a legal mandate for infertility counseling and supportive care in reproductive health policies and programs [10]. Prevention of infertility almost is a neglected issue in health systems [11]. Furthermore, cultural and social factors in some communities are barriers to access infertility services in health sectors [3]. In many countries, narrow and selective policies remain an obstacle to progress, which leads to shortages in coverage of infertility services as reproductive rights. Thus, providing comprehensive access for infertility services and implementing broad programs are important policies for many health systems and governments.

In spite of the increasing body of evidence on reproductive health policy, there has been a scarcity of framing analyses that focus on infertility policy processes in lower middle- and middle-income countries (LMIC) [12]. Accordingly, this study aimed was to compare the national infertility policies and to evaluate its comprehensiveness in seven selected low-, middle- and high-income countries with a focus on prevention, diagnosis, therapeutic management, supportive and rehabilitation care, to provide a description of gaps or confirmation of desirable policies in a given health system.

## Methods

As part of a larger study on infertility policy analysis based on Walt & Gilson framework [12], this was a comparative study of infertility policy in a number of selected countries in order to analyze these policies using the universal health coverage (UHC) framework. The choice and analyses of policy documents are explained in the following sections.

### Definition

The policy documents were defined as all formal records and reports that were written by national governments, national scientific communities and academic societies, national authorities and international organizations’ decisions, reports, plans and actions such as World Health Organization, World Bank, world health statistics, world development indicators and demographic and health survey. The type of evidence are as follows: provincial annual reports, core public health function/standards documents, health human resources (HHR), human resource planning (HRP) annual reports, business plan, commissioning policy documents, clinical guidelines, health profession legislation, and other public health (PH) reports such as competency development and leadership frameworks. Newspapers, online advertising sites, movie content, and marketing channels were not being included.

### Selection of countries

In order to select countries, after an initial search, we selected 14 countries that had available policy documents related to the three dimensions of universal health coverage that are prevention, treatment, and supportive care for infertility services. However, the final selection of countries was based on the following criteria:

1. Having adequate documents
2. Availability of documents without restrictions
3. Provided that documents are in English

**Fig. 1:**
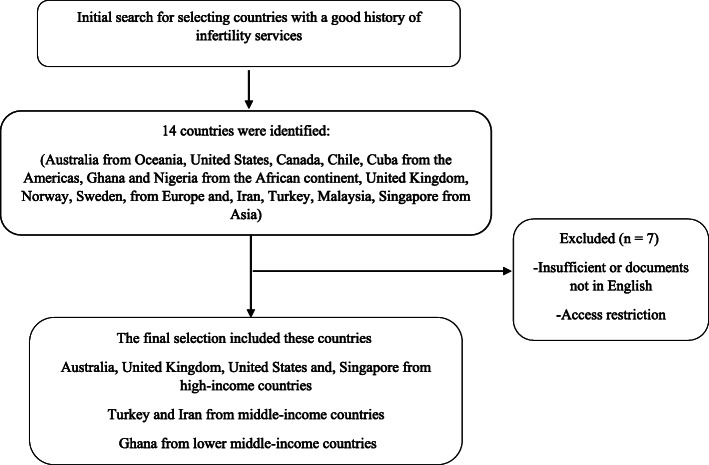
The flowchart of the selection of the countries

As such seven countries including Australia, the United Kingdom, the United States of America, Singapore, Turkey, Iran, and Ghana were selected. The flowchart of the selection of the countries is shown in Fig. 1.

### Selection of documents

**Fig. 2:**
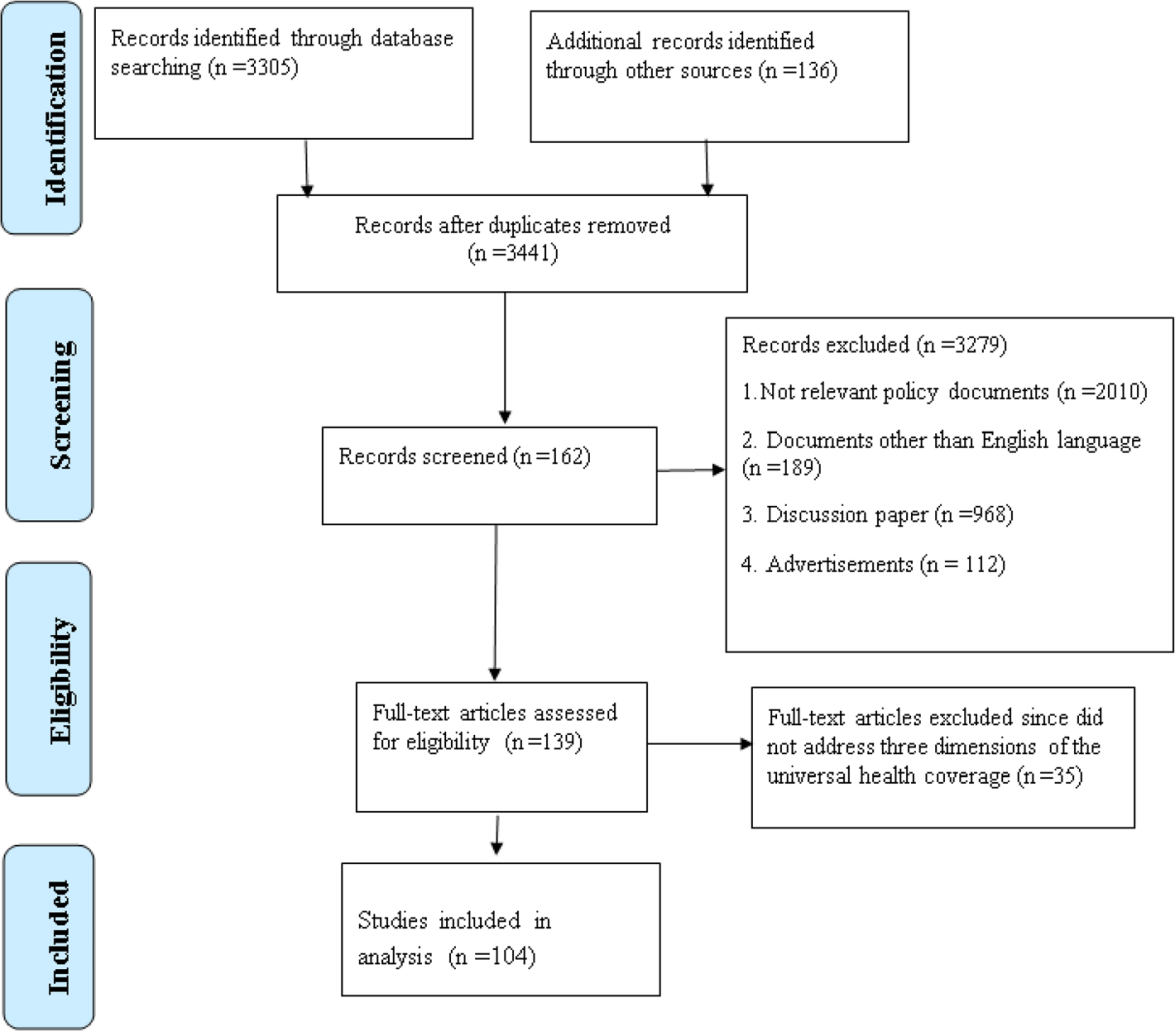
The study flowchart

An electronic systematic review search was conducted using PubMed, Scopus, ISI, Google Scholar, Google, all public websites, websites of health ministries, and websites of infertility clinics. Keywords included infertility, policy-making, affordability, accessibility, availability, acceptability, awareness, treatment, insurance, health policy, prevention, financial management, childlessness, equity, utilization, and cost. The search was conducted for each selected country. The search was limited to year 1994 to end of 2019. The year 1994 was chosen because since then the Cairo Conference has recommended countries to plan and implement action to prevent and treat infertility. Irrelevant documents were excluded. For instance, discussion papers, advertisements, and video clips were excluded. The study flowchart is shown in Fig. 2.

### Data extraction

A datasheet was prepared including the following information: name and aim of the policy, author(s) or organization name, and actors involved. These were tabulated and made ready for further analysis. Then, we identified three components for each policy document as defined by universal health coverage (UHC) in three scopes (prevention, treatment, and supportive care). These were: 1) financial protection 2) population coverage 3) and service package [13, 14].

### Scoring and analysis

Finally, the indicators and practical definition of policy components were provided and was finalized in two sessions by an expert panel consisting of two specialists in health policy, two public health scientists, a gynecologist who specialized in reproductive endocrinology and infertility and a reproductive health specialist. The explanatory variables and indicators are described in Table 1. Then a scoring manual for the three dimensions of universal health coverage that were related to the infertility policy for each selected country was presented. For each dimension, a description of the scoring is presented in Table 2.

**Table 1:** Definitions of dependent and explanatory variables

Variable	Measure
**Financial protection**	
Insurance coverage	The amount of risk or liability that is covered for an individual or entity by way of insurance infertility services
Government funding	Money provided by the government to pay for infertility services
Supply of voluntary and charitable donations	Financial support from voluntary associations or non-governmental organs
**Population coverage**	
Gender	Gender sensitivity in the service delivery^a^
Age	Availability of services for all age
Urban/rural coverage	Availability of infertility services in urban and rural
**Service package**	
Acceptability	Cultural, social and religious acceptance of infertility services
Accessibility/availability	Considering the number of service centers in relation to the population and their distribution
Awareness/registry	Having proper registry system
Preventive	Existing a preventive service which includes checkups, patient counseling and screenings to prevent infertility
Diagnostic and curative	Providing all infertility diagnostic and curative services
Rehabilitation, and supportive care	Existing rehabilitative infertility care includes empowerment of the couples to manage their conditions with proper counseling and enabling them to enjoy life by appropriate rules for adoption.

^a^ Having the following criteria: 1. Availability of infertility services to single women or men, widows, and homosexuals, 2. Equal treatment (e.g., waiting time, courtesy, privacy, information given) for male and female clients, 3. Facilities that are “male-friendly”

**Table 2:** Indicators that were used for the three dimensions and scoring

Financial protection	Not supportive	Moderate	Supportive
Insurance coverage /government funding/ supply of voluntary and charitable donations	One of the items	Two of the items	All three items
**Population coverage**	**Incomplete**	**Moderate**	**Complete**
Gender	Only for female or male	Married female and male	Gender sensitivity
Age	Limited	Reproductive age period	Not limited
Urban/rural coverage	Urban only without rural access	Urban with difficult access for rural	Urban with good access for rural
**Services features**	**Imperfect**	**Moderate**	**Perfect**
Acceptability	Low	Moderate	High
Accessibility /availability	Low	Moderate	High
Registry	Low	Moderate	High
Preventive services	Lack of policy	Poor policies	Efficient policies
Diagnostic and curative services	Lack of policy	Poor policies	Efficient policies
Rehabilitation, and supportive care services	Lack of policy	Poor policies	Efficient policies

## Results

### Country profiles

A profile of the selected countries is presented in Table 3. These included a range of information from the gross national income per capita to the fertility rate for each selected country. As shown, the prevalence of infertility rates ranged from 12 to 20% [15–21]. Comparing the cost of infertility treatment in countries showed that Singapore had the most expensive services for IVF treatment at 10000 to 15,000 USD for each cycle, but most of the costs (95%) are paid by the Medicare system [22]. On the contrary, Iran had the lowest IVF treatment price of 1272 USD per cycle [2].

**Table 3:** Country profile of the selected countries

	Lower middle- income	Middle-income		High-income			
	Ghana^a^	Iran	Turkey	UK	Australia	US (Federal government)	Singapore
**GNI per capita (USD)**^b^	4096	19,130	24,804	39,116	43,560	54,941	82,503
**Life expectancy (years)** ^b^	56.4	65.4	70.9	71.9	73.0	68.5	76.2
**Fertility rate**^b^	3.9	1.6	2	1.9	1.8	1.9	1.3
**HDI**^b^	0.592	0.798	0.791	0.922	0.939	0.924	0.932
**Current health expenditure (% of GDP)** ^b^	5.9	7.6	9.1	9.9	12.1	16.8	4.3
**Out-of-pocket expenditure (% of current health expenditure)** ^b^	36,105	39.66	16.95	14.79	19.558	11.08	36.74
**Prevalence of infertility (%)**	12–16	8–22.4	11.8–26.9	8–20 NHS =14	11–19.1	7–15	14.2–20
**The average cost of treating infertility (USD)**	From 4500	1272–2000	2800–5600	1965–5895	8000–10,000	12,400	10,000–15,000 governmental, 12,000–20,000 privet
**Estimated time and conditions for adopting policies**	1995	1987	1989	1951	1970	1944	1986
	With the beginning of the use of assisted reproductive technology	With the beginning of the use of assisted reproductive technology	With the beginning of the use of assisted reproductive technology	The first ideas about infertility treatment by artificial insemination	The first ideas about introducing IVF research	Once evidence of human fertilization in vitro	With the beginning of the use of assisted reproductive technology
**Political climate when policy is adopted**	Nothing important to report	Decrease TFR with pro-natalist context and increase in economic income	Reforms in health system and reproductive right	Access to new scientific findings for treatment	Access to new scientific findings for treatment	Access to new scientific findings for treatment	Decrease TFR with pro-natalist context

^a^ Ghana was a low-income country until 2007 and since then has been moved to the lower middle-income countries

^b^ Data is extracted from the World Bank website. https://data.worldbank.org 2015–2016

### Statistics

The flowchart is depicted in Fig. 1. In all 287 policy documents were retrieved for the selected countries, of which 183 irrelevant documents were excluded. The remaining 104 documents were examined for the components of universal health coverage, which are described in the following sections:

### Financial protection

The financial protection was inadequate in Ghana, and the USA, intermediate in Turkey and Iran, and good in Australia, the United Kingdom, and Singapore. For instance, all IVF clinics in Ghana are private, and there is no insurance coverage for infertility treatment. However, there are two voluntary and non-profit organizations that assist those who are at desperate need [23]. In the United States, there is no government service for the treatment of infertility. In only 15 states, the treatment of infertility is covered by private insurance [9, 24]. Specific limitations in the funding of Assistant reproductive technique (ART) were indicated for Turkey, such as offering funding based on fertility status that only primary infertility is covered by insurance [25]. In the UK, the National Health Service (NHS) provides infertility services and all patients have the right to be referred to an NHS hospital for the initial investigation, although waiting lists for treatment can be very long in some areas. In Australia and Singapore, the government provides infertility services under the Medicare system [22, 26, 27]. In Iran, insurance companies cover 20 to 80% of the cost of medical and diagnostic tests for infertility and pay fees for medications in primary and secondary infertility. Also, in the registry system, the government support package included two Intrauterine Insemination (IUI) and one IVF cycle in public centers has become free of charge since 2017 for couples who were eligible [28]. Table 4 presents the findings.

**Table 4:** Infertility services indicators in 7 selected countries

	Ghana	Iran	Turkey	UK	US	Australia	Singapore
**UHC dimensions**							
**Financial protection**	Not supportive	Moderate	Moderate	Supportive	Not supportive	Supportive	Supportive
**Population coverage**	Incomplete	Complete	Complete	Complete	Incomplete	Complete	Complete
**Service features**	Imperfect	Imperfect	Imperfect	Perfect	Perfect	Perfect	Perfect

### Population coverage

We examined infertility policies for converge to suggest whether infertility care covers both males and females, include different age groups, and offered to those who are living in urban or rural areas in the selected countries. In Ghana there is no governmental infertility care service at all. In addition, since there is a general belief in Africa (including Ghana) that men are not infertile; therefore, there is no adequate information about male infertility and related health services [29]. However, infertility services that are provide in the private sector for women, have no age limit. Iran and Turkey have comparable position on population coverage, such as providing infertility treatment for both men and women, and have age limit in the therapeutic process for women (less than 42 in Iran and 39 years in Turkey) in the governmental sector. In Iran, the government support package reserved for primary infertility and secondary infertility in a new marriage [30–32]. There was no evidence of difference in care provision between men and women. In the USA a growing body of literature shows that access to male infertility care is limited by several factors [33]. Most infertility clinics have age limitations for women (37 years or younger) [34]. Also the National Health Service in the UK provides infertility treatment for women with age limitation (up to three full cycles of IVF will be offered to eligible couples where the woman is aged between 18 and 39 and 1 cycle for where the woman is aged 40–42 years.) [35]. The service coverage that is urban /rural coverage were found to defer in the selected countries. The findings are presented in Table 4.

### Service package

As of 2015, in Ghana, only 14 private clinics are offering ART for nearly 6 million women in reproductive age (15-45 year). Unfortunately for the treatment of infertility, many women chose traditional healing and mediation [23, 29, 36]. The ART services are provided in 46 private and 44 public clinics in Iran, but none of them have registration systems or reporting transparent successful rates, most of which are in Tehran, the capital [25]. Currently, there are over 130 fertility clinics operating in Turkey. A number of fertility clinics are located in public or teaching hospitals, but most are private. They are mostly located in big urban areas [25]. There were 74,357 ART treatment cycles reported from Australia in 59 private physician clinics, 12 private hospital-based clinics, and 5 public clinics in 2016 [25, 37]. Based on CDC’s 2017 Fertility Clinic Success Rates Report, there were 284,385 ART cycles performed at 448 reporting clinics in the United States during 2017. These clinics are located throughout the country with a density in the west [38]. In Singapore, eleven infertility treatment centers are in the south [25]. All 78 infertility clinics in the UK are private or teaching hospitals [25] and the referral system in NHS that presented by a general physician are helpful for better accessibility and affordable services.

Impending health care reform for the Infertility Prevention Project (IPP) is working on multiple levels (federal, state, and local), within and across the USA, but there was no supportive care plan for infertile couples [39]. The Academy of Medicine (AMS) and the Ministry of Health (MOH) in Singapore have developed the clinical practice guidelines for assessment and management of infertility at the primary health care level for prevention proposes [22]. In the UK screening and prevention programs such as educational interventions are supported by the NHS and the Department of Health and Social Care [40]. Additionally, psychological, social, and financial support is provided by the NHS and volunteer groups [41]. Australian and New Zealand Infertility Counselors Association, Australian general practitioners, and Australia’s National Infertility Network have several educational programs for prevention and supportive care [26, 42–45]. In the other three countries (Ghana, Iran, and Turkey) we did not find any document for prevention or supportive care. The final score of the infertility policy situation of the selected countries is provided in Table 4.

## Discussion

This study analyzed data to compare infertility care policies among the selected countries representing lower middle-, middle-, and high-income countries including Ghana, Iran, Turkey, United Kingdom, Australia, United State of America, and Singapore. We analyzed the data based on the universal health converge framework including assessment of different aspects of its three dimensions that were financial protection, population converge, and services features.

The findings, generally, indicated that countries that are financially stable had better policies on infertility services. However, among rich countries, infertility care policy in the USA did not show a high score as expected. It is argued that the current situation in the USA might be due to the fact that infertility is not considered to be a disease by the USA government and thus support for those with infertility is limited. Similarly, in poor countries with limited financial resources where governments are struggling with the burden of infectious diseases, injuries, and high neonatal mortality and severe malnutrition, infertility services are very expensive and are not given priority [46–48]. Thus it is obvious that the use of assisted reproductive technologies for the treatment of infertility is an ongoing global reproductive health problem in both low and high income settings [2].

The other issue that should be discussed is the issue of equity and responsibility in the health system on infertility care. In countries in which infertility is not recognized as a medical condition or a human and reproductive right, not favorable comprehensive policies and services could be observed [33, 49, 50]. Such insufficient policies might cause limited financial protection for providing infertility services and perhaps would increase the financial burden to infertile couples and their family. Studies showed that there are considerable inequalities in access to effective treatments in countries such as the United States and Ghana [7, 29].

The role of population policies in addressing infertility programs is very important. After a prolonged Total Fertility Rate (TFR) decline due to family planning and socioeconomic factors in many developed and developing countries, some of these countries introduced pronatalist incentives [51]. As shown in Table 1, Australia, Singapore, and Iran have the lowest TFR among other countries. In these countries, strong pronatalist narrative policies have been formulated [51–53]. The justification for such policies is the fact that governments think support programs for infertility care could be a strategy to help to increase the population. For example, in Iran, the Ministry of Health is committed to infertility treatment and has mandated insurance companies to cover the cost of therapeutic and diagnostic tests and to subsidize the price of drugs for the treatment and has expanded the public infertility clinics [28].

Generally, progress in life expectancy, survival values, gender equity, and community values are viewed in countries that have a favorable condition in UHC on infertility services. This emphasis the hypothesis that if human rights, life expectancy, and survival were valuable in a population, then appropriate policies for UHC will possibly be presentable and there may be forceful mechanisms for agenda-setting in emerging conditions such as infertility [54]. In addition, attention to infertility services is very much related to the development goals of countries. For example, in Japan with 2,400,000 infertile individuals [55], presence of numerous infertility clinics (518 clinics in 2016), the existence of adequate laws and protocols related to infertility services, the existence of full insurance and government funding, show the importance of increasing the youth population for economic activity and development [25].

Social concerns about the use of third-party involvement in ART could be a barrier for acceptability and service coverage [23, 28, 56]. Additionally, major challenges are seen in providing preventive and supportive infertility care in communities where cultural, religious, and social complexity exists. On-demand side, infertility related stigma in many societies has led to the reduction in seeking supportive care services [57, 58], sexual health education, and STI care, which are the main foundations for the prevention of infertility [59, 60].

Neglecting infertility in the long term can be lead to population decline and aging. It is argued that this could be happen due to several reasons including the decrease in timely marriage, and fewer new births. However, these by itself can increase the burden of health care systems. Likewise, remaining untreated infertility or failure of preventing it, might double the cost of the health care system [61].

Responsiveness to equity in allocating financial resources to infertility, which are related to future economic development and growth, is important in lower middle- and middle-income countries with limited national resources. So, by using appropriate insurance coverage, efficient resource allocation strategies in health care and adequate funding strategies, it is possible to reduce the share of out-of-pocket payments and equity in allocating financial resources for it [62]. Therefore, paying attention to the implementation of appropriate policies to prevent infertility, timely treatment and rehabilitation of couples for proper childbearing, can be an appropriate policy to invest in creating a productive and economically active generation to grow and improve Gross Domestic Product (GDP) in the future.

However, our study had several limitations, for example, in some countries, such as Turkey, some policy documents and programs were not available in English. Also, all documentation in many countries might not be online and therefore inadequate access to the national documents and lack of comparable studies was the main limitations.

## Conclusion

The findings indicated that the provision of infertility care services varied among high, middle, and lower middle income countries as expected and depended on many factors including effective access to healthcare services as well as socio-economic and cultural issues. However, the findings showed that income is not the only factor that fulfills universal health coverage for infertility care services. Perhaps to achieve equity in infertility care it should be seen as a universal human right. Additionally, in lower middle and middle income countries, prevention plans of infertility could be integrated with primary health care for promoting infertility services.

## Data Availability

The data will be available on a reasonable request from the corresponding author in due course.

## Abbreviations

ART: Assistant Reproductive Technology
CDC: Centers for Disease Control and Prevention
GNI: Gross National Income
GDP: Gross Domestic Product
HR: Health Human Recourse
HRP: Human Reproduction Programme
ICPD: The International Conference on Population and Development
IPP: Infertility Prevention Project
IUI: Intrauterine Insemination
IVF: In Vitro Fertilization
LMIC: Lower middle and Middle Income Countries
NHS: National Health Service
PH: Public Health
SRHR: Sexual and Reproductive Health and Right
TFR: Total Fertility Rate
WHO: World Health Organization
